# High‐resolution computed tomography for the prediction of mortality in acute respiratory distress syndrome: A retrospective cohort study

**DOI:** 10.1002/hsr2.418

**Published:** 2021-10-08

**Authors:** Ryosuke Imai, Naoki Nishimura, Osamu Takahashi, Tomohide Tamura

**Affiliations:** ^1^ Department of Pulmonary Medicine Thoracic Center, St. Luke's International Hospital Tokyo Japan; ^2^ Graduate School of Public Health St. Luke's International University Tokyo Japan

**Keywords:** acute lung injury, acute respiratory distress syndrome, ARDS mortality, chest high‐resolution computed tomography, diffuse pattern

## Abstract

**Background and Aims:**

Acute respiratory distress syndrome **(**ARDS) demonstrates several image patterns on high‐resolution computed tomography (HRCT). The purpose of this study was to investigate the relationship between specific HRCT findings and the prognosis of ARDS.

**Methods:**

This was a retrospective cohort study performed in a single hospital in Japan. We categorized HRCT findings into three distribution patterns: diffuse, subpleural sparing, and dorsal patterns. All patterns were assessed at three levels of each lung. Multivariable logistic regression analysis was used to identify parameters associated with in‐hospital mortality.

**Results:**

A total of 144 patients with ARDS (age: 72 ± 16 years, 112 men) were included in the study. The in‐hospital mortality rate was 42% (survivors, n = 83; nonsurvivors, n = 61). Nonsurvivors were significantly older (70 ± 17 vs 76 ± 13, *P* = 0.01) and had lower serum albumin levels (*P* = 0.01), more traction bronchiectasis (*P* = 0.02), and more diffuse pattern (*P* < 0.001) than survivors. The presence of diffuse patterns was an independent adverse prognostic factor for predicting mortality (odds ratio, 1.32; 95% confidence interval [CI]: 1.08‐1.61, *P* = 0.007).

**Conclusions:**

HRCT distribution patterns may predict mortality in ARDS patients.

**Abbreviations**
APACHE: acute physiology and chronic health evaluation
ARDS: acute respiratory distress syndrome
CI: confidence interval
CRP: C‐reactive protein
DAD: diffuse alveolar damage
HRCT: high‐resolution computed tomography
KL‐6: Krebs von den Lungen‐6
LDH: lactate dehydrogenase
OR: odds ratio
SD: standard deviation
WBC: white blood cell

## 1 INTRODUCTION

Acute respiratory distress syndrome (ARDS) is characterized by an acute onset of hypoxemia with bilateral pulmonary infiltrates found on chest radiography, secondary to underlying disorders associated with lung injury caused by nonspecific hyperinflammation with neutrophils. Despite advances in the care of critically ill patients, the mortality rate of ARDS remains high.^1^, ^2^, ^3^

High‐resolution computed tomography (HRCT) of the chest is not routinely performed to diagnose or manage patients with ARDS.^4^ However, the Berlin definition recognizes the use of CT instead of chest radiography for the detection of lung opacities.^1^ Several studies have also demonstrated that HRCT can assess the pathophysiological condition and mortality of ARDS patients.^5^, ^6^ Ichikado et al, using a validated HRCT scoring system, reported that fibroproliferative changes in the lung were associated with increased mortality.^5^ Similarly, Chung et al indicated that more extensive lung involvement and traction bronchiectasis on CT were associated with a higher risk of death.^6^

While most patients with clinical ARDS have histological evidence of diffuse alveolar damage (DAD), ARDS can present with other histological patterns, including organizing pneumonia and acute fibrinous and organizing pneumonia. Moreover, HRCT radiological patterns vary according to histological patterns.^7^, ^8^ HRCT in patients with DAD shows a diffuse crazy‐paving pattern or dorsal pattern with a gravitationally dependent gradient.^8^ In contrast, sparing of the subpleural area of the lungs can be seen with organizing pneumonia.^9^

However, limited data are available on the prevalence, clinical implications, and association of the HRCT radiological patterns with the prognosis of ARDS. Furthermore, the HRCT radiological patterns that reflect active fibroproliferative changes most accurately are unknown.

The purpose of this study was to examine the relationship between HRCT radiological patterns and ARDS prognosis.

## 2 METHODS

### 2.1 Subjects

This was a retrospective cohort study conducted at St. Luke's International Hospital in Japan. Patients diagnosed with ARDS according to American‐European Consensus Conference Criteria from 1 April 1, 2004, to March 31, 2016, at our hospital were included. Eligible patients were adults (≥16 years of age) who underwent HRCT at diagnosis. We excluded patients with chronic interstitial lung disease and congestive heart failure, patients recovering from cardiopulmonary arrest, and those with life expectancy less than two months due to advanced neoplasms. The study was approved by the Institutional Review Board of St. Luke's International Hospital (institutional review board no. 17‐R039).

### 2.2 Data collection

Medical records were reviewed retrospectively for demographic information (age, sex), underlying illness, presumed cause of ARDS, APACHE II score, PaO_2_/FiO_2_, ventilator settings at diagnosis, laboratory and radiological data, medical treatment, corticosteroid use, and clinical outcomes.

### 2.3 HRCT assessment

**FIGURE 1:**
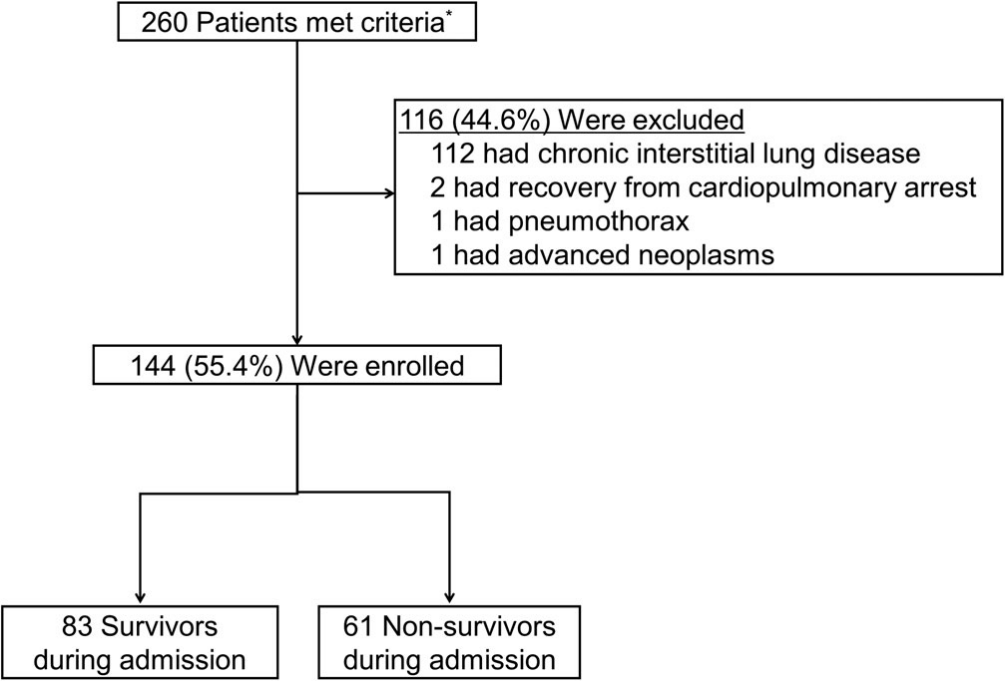
High‐resolution computed tomography (HRCT) distribution patterns. Examples of typical computed tomography (CT) images from our cases. A, The subpleural sparing pattern was defined as an area with more than 50% of the sparing area adjacent to the pleura in the presence of infiltrates in the lung field. B, The dorsal pattern as an area with infiltrates occupying less than 50% of the dorsal part of the lung. C, The diffuse pattern was defined as an area with more than 50% of infiltrates spreading into the pleura in a certain slide from the CT

In our facility, all patients suspected of ARDS underwent whole lung volumetric HRCT scanning of the chest at diagnosis using a multidetector‐row CT scan to rule out pleural effusion or lung collapse. The HRCT scans were evaluated by two independent observers blinded to the patients' clinical characteristics and study outcomes. Disagreements between the observers were resolved by consensus. We categorized HRCT findings into three distribution patterns: diffuse, subpleural sparing, and dorsal (Figure 1). The diffuse pattern was defined as an area with more than 50% of infiltrates spreading into the pleura in a certain slide from the CT. The subpleural sparing pattern was defined as an area with more than 50% of the sparing area adjacent to the pleura in the presence of infiltrates in the lung field, and the dorsal pattern as an area with infiltrates occupying less than 50% of the dorsal part of the lung. When multiple patterns were exhibited, we gave priority to a diffuse pattern. The presence of traction bronchiectasis was also assessed. All findings were assessed in six HRCT slices at three levels of the lungs. The upper level was the area of the lung 1 cm above the superior margin of the aortic arch, the middle level was the area 1 cm below the level of the carina, and the lower level was the area 1 cm above the top of the right diaphragm. The total number of slices and the number of slices with the three distribution patterns were estimated.

### 2.4 Statistical analyses

Data are represented as mean ± standard deviation (SD) for continuous variables and frequencies (percentage) for categorical variables. Categorical variables were compared using the Chi‐square test or Fisher's exact test. Continuous variables were compared using the Student's *t*‐test. To identify independent risk factors for in‐hospital mortality, prognostic factors, such as APACHE II score, all significant variables of univariate analyses were included in the multivariable logistic regression model. All statistical tests were two‐tailed, and significance was accepted at *P* ≤ 0.05. All analyses were performed using SPSS for Windows, version 22.0.

## 3 RESULTS

**FIGURE 2:**
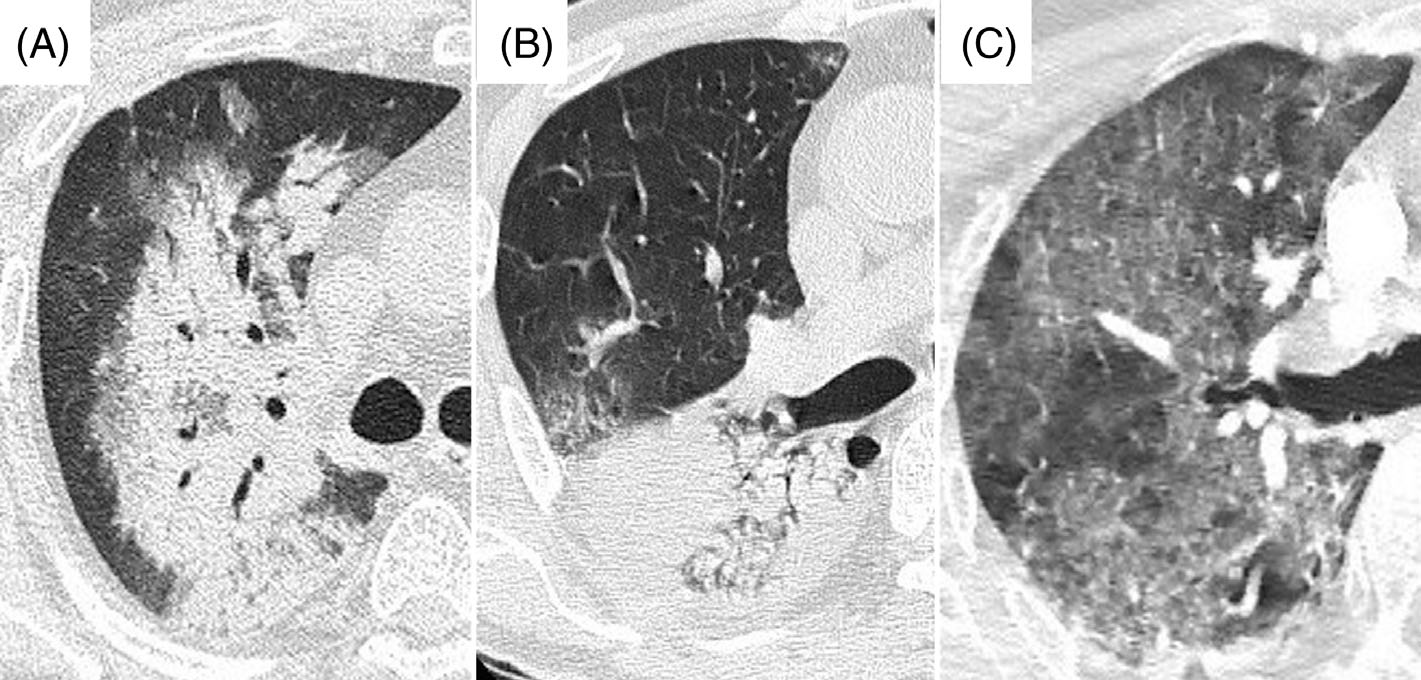
Outline of the study. ^*^All patients with respiratory failure suspected of ARDS underwent chest CT, and those suspected of infectious pneumonia on CT were excluded. One hundred and twelve patients were ineligible because of chronic interstitial lung diseases, two patients recovered from cardiopulmonary arrest, and one had pneumothorax and another one had advanced neoplasms and was expected to live for less than 2 months. ARDS, acute respiratory distress syndrome; CT, computed tomography

One hundred and forty‐four patients (mean age ± SD, 72 ± 16 years, 112 [78%] men) met the inclusion criteria (Figure 2). Their in‐hospital mortality rate was 42%. Fifty‐five patients (38%) had sepsis resulting in ARDS, 34 patients (24%) had pneumonia, 18 patients (13%) had aspiration, and 11 patients (8%) had drug‐induced pneumonitis. Patient characteristics, laboratory data, presence of mosaic appearance, and traction bronchiectasis were compared between survivors and nonsurvivors (Table 1). Nonsurvivors were significantly older (mean 70 ± 16 vs 76 ± 13, *P* = 0.01) and had lower serum albumin levels (mean 3.0 ± 0.7 vs 2.7 ± 0.6 g/dL, *P* = 0.01) compared to survivors. No significant differences were seen in other clinical conditions.

**TABLE 1:** Demographic and clinical characteristics of survivors and nonsurvivors

Characteristic	Total n = 144	Survivors (n = 83, 58%)	Nonsurvivors (n = 61, 42%)	*P*‐value^a^
Age, (years)	72 ± 16	70 ± 17	76 ± 13	0.01
Male, n (%)	112 (78)	64 (77)	48 (79)	>0.99
PaO_2_/FiO_2_	173 ± 38	171 ± 40	178 ± 32	0.37
APACHE II score	22 ± 6	23 ± 7	22 ± 6	0.70
Ventilator use, n (%)	90 (60.3)	50 (55.6)	40 (44.4)	
Cause of lung injury, n (%)				
Sepsis	55 (38)	43 (52)	12 (20)	
Pneumonia	34 (24)	12 (14)	22 (36)	
Aspiration	18 (13)	10 (12)	8 (13)	
Drug induced	11 (8)	8 (10)	3 (5)	
Other	26 (18)	10 (12)	16 (26)	
Time from diagnosis (d)	0.4 ± 1.0	0.2 ± 0.9	0.6 ± 1.1	0.06
Laboratory data				
WBC (per mm^3^)	12 084 ± 6745	12 533 ± 6285	11 470 ± 7336	0.45
CRP (mg/dl)	13.2 ± 10.0	13.3 ± 11.2	13.1 ± 8.2	0.88
Albumin (g/dl)	2.9 ± 0.7	3.0 ± 0.7	2.7 ± 0.6	0.01
pH	7.41 ± 0.11	7.41 ± 0.12	7.42 ± 0.11	0.53
HCO_3_^−^ (mmol/l)	23.6 ± 4.0	23.5 ± 4.3	23.8 ± 3.7	0.67
Lactate (mmol/l)	2.1 ± 2.0	2.3 ± 2.3	1.9 ± 1.5	0.44
LDH (U/l)	421 ± 319	426 ± 372	412 ± 234	0.82
KL‐6 (pg/ml)	831 ± 1339	655 ± 721	1011 ± 1748	0.06
HRCT findings, n (%)				
Traction bronchiectasis	66 (44.9)	31 (47.0)	35 (53.0)	0.02

*Note*: Continuous variables are expressed as mean ± SD, and categorical data are expressed as n (%).

Abbreviations: APACH, Acute Physiology and Chronic Health Evaluation; CRP, C‐reactive protein; KL‐6, Krebs von den Lungen‐6; LDH, lactate dehydrogenase; WBC, white blood cell.

^a^ Comparison between survivors and nonsurvivors.

Table 2 presents HRCT findings for the survivors and non‐survivors. Interobserver reliability for decision of the distribution patterns in the upper, middle, and lower levels had κ values of 0.76, 0.78, and 0.73, respectively. The nonsurvivors had more involved lung slices (mean, 5.5 ± 0.9 vs 5.2 ± 1.2, *P* = 0.07), more bronchiectasis (35 [53%] vs 31 [37%], *P* = 0.02), and a larger number of slices with diffuse pattern (mean, 4.4 ± 1.7 vs 3.1 ± 2.1, *P* < 0.001) than survivors.

**TABLE 2:** High‐resolution computed tomography (HRCT) findings in survivors and nonsurvivors

Characteristic	Survivors (n = 83, 58%)	Nonsurvivors (n = 61, 42%)	*P*‐value^a^
Affected lung slices	5.2 ± 1.2	5.5 ± 0.9	0.07
Traction bronchiectasis, n (%)	31 (37)	35 (53)	0.02
Diffuse pattern slices	3.1 ± 2.1	4.4 ± 1.7	<0.001
Subpleural sparing pattern slices	1.5 ± 1.8	0.5 ± 1.1	0.56
Dorsal pattern slices	0.5 ± 1.3	0.6 ± 1.1	0.94

*Note*: Continuous variables are expressed as mean ± SD, and categorical data are expressed as n (%).

^a^ *P‐*values comparing survivors and nonsurvivors.

**FIGURE 3:**
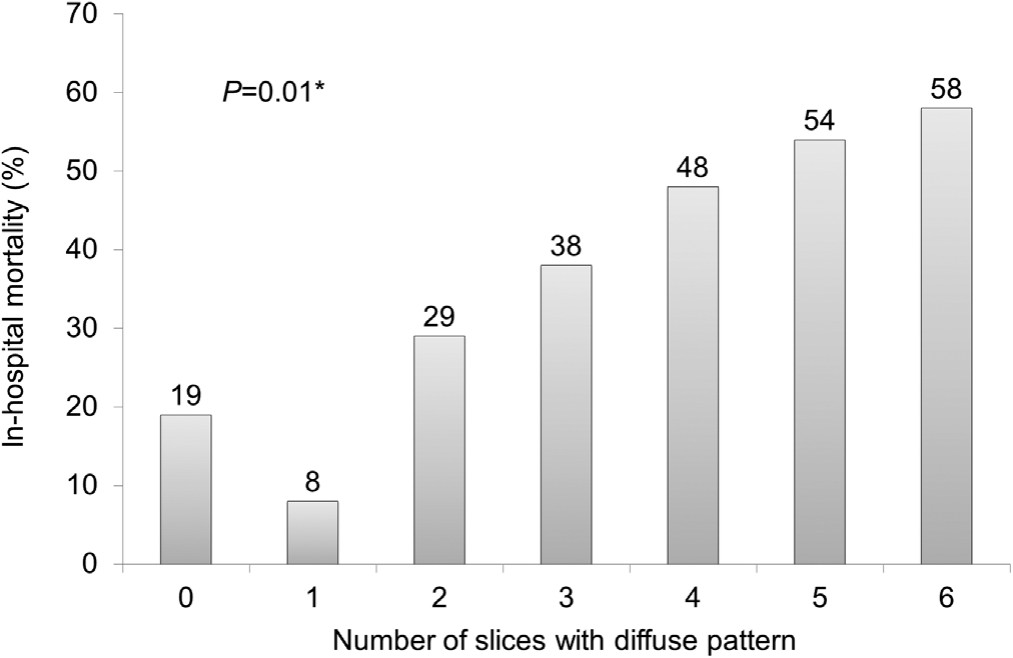
In‐hospital mortality rate for the number of slices with diffuse patterns ^*^Chi‐square test

In the multivariable logistic regression analysis, we created two models. The first model included age, serum albumin, APACHE II, traction bronchiectasis, and number of involved lung slices. In the second model, the number of involved lung slices was replaced by the number of slices with diffuse pattern. The number of slices with diffuse pattern was an independent adverse prognostic factor (odds ratio 1.32, 95% CI, 1.08‐1.61, *P* = 0.007). In contrast, there was no correlation between the number of involved lung slices and mortality (odds ratio 1.11, 95% CI, 0.75‐1.64, *P* = 0.61) (Table 3). Figure 3 shows the relationship between the number of slices with diffuse pattern and in‐hospital mortality. Larger number of slices with the diffuse pattern were associated with higher mortality rate.

**TABLE 3:** Multivariate logistic regression analysis for prognostic factors associated with in‐hospital mortality

Model 1	OR (95% CI)	*P*‐value
Age	1.03 (1.00‐1.06)	0.03
Serum albumin	0.61 (0.31‐1.07)	0.08
APACHE II	0.97 (0.91‐1.03)	0.27
Traction bronchiectasis	1.62 (0.77‐3.40)	0.20
Affected lung slices	1.11 (0.75‐1.64)	0.61
Model 2		
Age	1.03 (1.00‐1.05)	0.06
Serum albumin	0.60 (0.32‐1.14)	0.12
APACHE II	0.97 (0.91‐1.03)	0.31
Traction bronchiectasis	1.32 (0.62‐2.83)	0.47
Diffuse pattern slices	1.32 (1.08‐1.61)	0.007

Abbreviation: APACHE, Acute Physiology and Chronic Health Evaluation.

## 4 DISCUSSION

In this study, we demonstrated a correlation between distribution patterns on HRCT and prognosis in patients with ARDS. Diffuse patterns were an independent prognostic factor for in‐hospital mortality. Previous studies have examined the prognostic factors for ARDS using HRCT. Chung et al suggested that more extensive lung involvement and bronchiectasis predicted mortality in ARDS.^6^ Ichikado et al reported that the extent of fibrosis determined using a validated scoring system for HRCT predicted ARDS mortality.^5^ To our knowledge, the relationship between distribution patterns on HRCT and ARDS prognosis has not been reported. Distribution patterns may aid in identifying patients who can recover quickly and completely with adequate treatment.^4^

ARDS is a heterogeneous disease that exhibits different patterns on HRCT. Several studies have demonstrated that DAD, including hyaline membranes and the presence of fibrosis, are pathological hallmarks of ARDS.^7^, ^10^ ARDS can also present with other histological patterns including organizing pneumonia and acute fibrinous and organizing pneumonia. Moreover, HRCT radiological patterns vary according to histological patterns.^7^, ^8^

Previous studies have reported that in the acute stage of DAD, alveolar edema with exudation of the plasma into the alveolar spaces develops due to inflammatory injury, followed by organization and fibrosis in the late phase of DAD.^4^ On HRCT, the acute phase of DAD manifests as diffuse patchy ground‐glass opacities with areas of consolidation and septal thickening, and focal areas of spared, normal attenuation, creating a geographic appearance. Microscopically, evidence of lung injury is present even in this subpleural sparing area.^9^ In the late phase of DAD, consolidation spreads into the pleura since DAD progresses from the exudative phase to the proliferative phase in the normally aerated subpleural lung area.^9^ Some studies have reported that other typical HRCT presentations of DAD include an increasing parenchymal density gradient from the ventral to the dorsal lung.^11^, ^12^, ^13^, ^14^ The dorsal pattern may have a large and normally aerated lung area, or the exudative phase of DAD may manifest as a small fibroproliferative lung area. Organizing pneumonia is also known as one of the histologic findings of ARDS and presents with various patterns of diffuse parenchymal abnormalities on HRCT. Bilateral consolidation is predominantly peribronchial, with sparing of the subpleural area of the lung along with thickened septa.^9^

Our study suggests that ARDS patients with a diffuse pattern in which the infiltrates spread to the pleura have a higher mortality. In the acute phase of DAD, the anterior or subpleural lung area with a microscopically exudative phase of DAD appears to be spared as normal attenuation. On the contrary, in the late stage, DAD progresses to the fibroproliferative phase, and consolidation extends to the pleura. In other words, the diffuse pattern may represent the fibroproliferative phase of DAD, which is a more advanced phase with more organization or fibrosis.

Another possibility could be that subpleural sparing is observed if congestion or edema is significant. Edema in the subpleural lung area is likely to be drained because the peripheral vessels are small in caliber and easily adapted for vasoconstriction and vasodilatation, which alters the peripheral resistance to flow from the larger arteries. Furthermore, there are numerous precapillary anastomoses between the pulmonary arteries and veins in which shunting may occur.^15^ Therefore, a subpleural sparing pattern may be associated with a better prognosis than a diffuse pattern because it is more affected by congestion or edema.

Our study demonstrated that patients with a greater diffuse pattern tended to have higher KL‐6 levels and more traction bronchiectasis than those with subpleural sparing and dorsal patterns. KL‐6 is classified as a human MUC1 mucin protein. The regenerating type II pneumocytes are the primary cellular source of KL‐6/MUC1. KL‐6/MUC1 is one of the key molecules involved in the intra‐alveolar fibrotic process and pulmonary fibrosis.^16^, ^17^ These findings are indicative of a more advanced fibroproliferative response in patients with a diffuse pattern.

The present study has limitations. First, the distribution patterns from HRCT were obtained at the undefined stages of ARDS progression. These patterns may change over the clinical course of the disease and therefore may not predict ultimate mortality. However, in most cases, HRCT was performed at the time of ARDS diagnosis. Hence, the HRCT scan reviewed in this study can be regarded as the image at the time of treatment initiation. Appropriate therapeutic intervention initiated at an earlier phase of DAD is likely to lead to a better prognosis. Second, our patients did not receive consistent treatment. Corticosteroids were not administered to any patient. The effects of corticosteroids on survival in ARDS remain controversial, and there are no data to support that corticosteroids improve mortality.^18^ Furthermore, positive end‐expiratory pressure (PEEP) was not used in all patients. Thus, we were unable to evaluate the effect of PEEP on the distribution patterns.^14^ A prospective study with uniform settings and treatment is needed to confirm our findings.

In conclusion, we demonstrated that in patients with ARDS, HRCT distribution patterns may predict mortality, disease activity, and the phase of fibroproliferative changes in the lungs.

## CONFLICT OF INTEREST

The authors declare that there are no known conflicts of interest associated with this publication.

## FINANCIAL/NONFINANCIAL DISCLOSURES

There has been no financial support for this work that could have influenced its outcome.

## AUTHOR CONTRIBUTIONS

Conceptualization: Ryosuke Imai and Naoki Nishimura.

Formal analysis: Ryosuke Imai, Naoki Nishimura and Osamu Takahashi.

Investigation: Ryosuke Imai, Naoki Nishimura and Tomohide Tamura.

Writing—Original Draft Preparation: Ryosuke Imai.

Writing—Review & Editing: Ryosuke Imai, Naoki Nishimura, Osamu Takahashi, and Tomohide Tamura.

All authors have read and approved the final version of the manuscript.

Ryosuke Imai confirm that they had full access to all of the data in the study and take complete responsibility for the integrity of the data and the accuracy of the data analysis.

## TRANSPARENCY STATEMENT

The lead author affirms that this manuscript is an honest, accurate, and transparent account of the study being reported; that no important aspects of the study have been omitted.
